# Transient expression of fluorescent proteins and Cas nucleases in Phytophthora agathidicida via PEG-mediated protoplast transformation

**DOI:** 10.1099/mic.0.001547

**Published:** 2025-03-28

**Authors:** Max Hayhurst, Jochem N. A. Vink, Maxence Remerand, Monica L. Gerth

**Affiliations:** 1 School of Biological Sciences, Victoria University of Wellington, Wellington 6012, New Zealand

**Keywords:** kauri dieback, molecular biology, oomycete, PEG/CaCl_2_, *Phytophthora*, protoplasts, transformation

## Abstract

*Phytophthora* species are eukaryotic plant pathogens that cause root rot and dieback diseases in thousands of plant species worldwide. Despite their significant economic and ecological impacts, fundamental molecular tools such as DNA transformation methods are not yet established for many *Phytophthora* species. In this study, we have established a PEG/calcium chloride (CaCl_2_)-mediated protoplast transformation method for *Phytophthora agathidicida*, the causal agent of kauri dieback disease. Adapting a protocol from *Phytophthora sojae*, we systematically optimized the protoplast digesting enzymes, recovery media composition and pH. Our findings reveal that chitinases are essential for *P. agathidicida* protoplast formation, and the optimum pH of the recovery medium is 5. The media type did not significantly impact protoplast regeneration. Using this protocol, we generated transformants using three plasmids (i.e. pTdTomatoN, pYF2-PsNLS-Cas9-GFP and pYF2-PsNLS-Cas12a-GFP), which expressed fluorescent proteins and/or Cas nucleases. The transformants were unstable unless maintained under antibiotic selective pressure; however, under selection, fluorescence was maintained across multiple generations and life cycle stages, including the production of fluorescent zoospores from transformed mycelia. Notably, we observed the expression of GFP-tagged Cas nucleases, which is promising for future CRISPR-Cas genome editing applications. This study demonstrates that *P. agathidicida* is amenable to PEG/CaCl_2_-mediated protoplast transformation. Although the resulting transformants require antibiotic selective pressure to remain stable, this transient expression system can be valuable for applications such as cell tracking, chemotaxis studies and CRISPR-Cas genome editing. The protocol also provides a foundation for further optimization of transformation methods. It serves as a valuable tool for exploring the molecular biology of *P. agathidicida* and potentially other closely related *Phytophthora* species.

## Introduction

*Phytophthora* is a genus of eukaryotic microorganisms that encompasses some of the most devastating plant pathogens in agriculture and native ecosystems [1]. A crucial method for molecular biology research is transformation – the uptake of exogenous genetic material into cells. Transformation has been a valuable tool for studying *Phytophthora* at the molecular level, enabling the generation of fluorescently labelled strains [2], gene silencing [3,4] and CRISPR-Cas genome editing [5,7]. Several different methods are used to transform *Phytophthora* species: PEG/calcium chloride (CaCl_2_)-mediated protoplast transformation [8], zoospore electroporation [9], *Agrobacterium tumefaciens*-mediated transformation [10] and microprojectile bombardment [11]. However, transformation efficiencies can be low, and protocols often require significant optimization for each species [12,14].

Kauri dieback disease, caused by *Phytophthora agathidicida*, is a devastating root and collar rot affecting New Zealand kauri trees (*Agathis australis*) [15]. *P. agathidicida* belongs to *Phytophthora* Clade 5, a group with an eastern Asian and Pacific centre of diversity [16]. Although kauri dieback symptoms were identified as early as the 1970s, *P. agathidicida* was only recognized as a new species in 2015 [16,17]. Clade 5 *Phytophthora* species, including *P. agathidicida*, have significant ecological and economic impacts; they affect important crop trees, such as cocoa and rubber, and ecologically vital species, like kauri [16,18]. The destruction of kauri forests by *P. agathidicida* not only threatens biodiversity but also impacts cultural and economic values associated with these iconic trees [15,19]. Despite their importance, *Phytophthora* Clade 5 is a poorly studied group, and molecular tools for studying Clade 5 species remain limited. While a chromosome-level genome sequence has recently become available for *P. agathidicida* [20], there are currently no established transformation methods for *P. agathidicida* (or any Clade 5 *Phytophthora* species). This lack of genetic tools poses a significant challenge for molecular biological investigations into this devastating group of pathogens, highlighting the need for transformation protocols.

Transformation of protoplasts – wall-less cells obtained by enzymatic digestion of hyphae – was the first successful method reported for *Phytophthora* [8,21], and it remains commonly used today [13,14, 22, 23]. The general steps of protoplast transformation protocols are protoplast production, transformation, recovery and selection. Protoplasts are typically obtained by treating hyphae with enzyme mixtures containing cellulases (β−1,4-glucanase) and β−1,3-glucanases [2,6, 8]. During the transformation process, protoplasts are suspended in osmotically stabilized buffers containing mannitol. DNA delivery is facilitated by PEG and CaCl_2_, sometimes in combination with cationic liposome complexes containing the DNA [8,24]. After incubation with DNA, a recovery medium is added to the protoplasts, typically a complex vegetable-based media supplemented with mannitol [2,6, 8]. Following recovery, protoplasts are typically selected using antibiotic resistance markers. Variations of this method have been successfully applied to numerous *Phytophthora* species [6,23, 25, 26].

This study aimed to establish a method for PEG/CaCl_2_-mediated protoplast transformation of *P. agathidicida*, adapting an existing protocol for *Phytophthora sojae* [6]. We optimized the digestion and recovery conditions; the resulting method is presented here. Overall, this work provides a reliable method for the transformation of *P. agathidicida*, facilitating further research into its biology and pathogenicity.

## Methods

### Materials

The Supplementary Information (available in the online version of this article) provides detailed protocols and references for media and transformation reagents. Unless otherwise noted, chemicals were from Sigma-Aldrich, and media were from BD Difco.

### Growth and maintenance of *P. agathidicida*

*P. agathidicida* isolate NZFS 3770 was obtained from Scion (Rotorua, New Zealand). *P. agathidicida* was initially cultured on selective cornmeal agar plates containing 250 µg ml^−1^ ampicillin (GoldBio), 100 µg ml^−1^ pentachloronitrobenzene, 0.001 % w/v pimaricin and 10 µg ml^−1^ rifampicin as described in *Phytophthora Diseases Worldwide* [27]. After selection, *P. agathidicida* was maintained by serial culture on 20% (w/v) pea agar at 22 °C [12] in the dark. New cultures were inoculated by transferring a 6 mm agar plug from the leading edge of the mycelium to a fresh growth medium.

### Plasmids for transformation

The pTdTomatoN plasmid [28] and pYF2-PsNLS-Cas12a-GFP [5] were kindly provided by Howard Judelson (University of California, Riverside). The pYF2-PsNLS-Cas9-GFP plasmid [6] was kindly provided by Felipe Arredondo (Oregon State University). PsNLS refers to the *P. sojae* nuclear localization signal [6].

### Protoplast isolation

Protoplasts were produced using a method adapted from Fang and Tyler [6]. Cultures were started by inoculating sterile 250 ml flasks containing 50 ml pea broth with five 6 mm agar plugs from the leading edge of mycelium on a pea agar plate of *P. agathidicida* 3770.

Cultures were grown in the dark for 72 h without shaking. Before harvesting cultures, the enzyme mixtures to be tested (Tables1  2) were prepared fresh in buffer (20 mM 2-(*N*-morpholino)ethanesulfonate, 0.4 M d-mannitol, 20 mM KCl, 10 mM CaCl_2_, pH 5.7) and sterile-filtered. Mycelia from the pea broth cultures were collected in 100 µm EASYstrainer cell strainers (Greiner Bio-One), washed with sterile water and subsequently left in 0.8 M d-mannitol for 20 min at 22 °C. After draining the liquid, the mycelial mats were transferred to one 15 ml tube containing the appropriate enzyme mixture (3 ml per plug). The tube was then incubated horizontally in a rotary shaker at 40 r.p.m. and 22 °C for variable lengths of time. For routine transformations, the mycelia were digested for 45 min. For the optimization experiments with the chitinase-containing enzyme mixture (Table 2)**,** the digestions were monitored for up to 3 h, and images were taken every 30 min to monitor the production of protoplasts.

**Table 1.** Original lysing enzyme mixtures. Three solutions (A, B, C) with different ratios of cellulase versus Lysing Enzyme were tested. The final enzyme concentration was 15 mg ml^−1^ in all solutions

Reagent	Solution A	Solution B	Solution C
Cellulase from *Trichoderma* sp. Sigma Aldrich C1794	10 mg ml^−1^	7.5 mg ml^−1^	5 mg ml^−1^
Lysing enzymes from *Trichoderma harzianum*Sigma Aldrich L1412	5 mg ml^−1^	7.5 mg ml^−1^	10 mg ml^−1^

**Table 2.** Chitinase-containing lysing enzyme mixture

Reagent	Final concentration
Cellulase from *Trichoderma* sp.Sigma Aldrich C1794	5 mg ml^−1^
β-glucanases from *Trichoderma longibrachiatum*Sigma Aldrich G4423	10 mg ml^−1^
Chitinase from *Trichoderma viride*Sigma Aldrich C8241-25UN	0, 0.08 or 0.17 U* ml^−1^

**One standard activity unit (U) is defined by the manufacturer as the quantity that liberates 1.0 mg of *N*-acetyl-d-glucosamine from chitin per hour at pH 6.0 at 25 °C in a 2 h assay.

The digested protoplasts were collected by sequentially filtering the suspension through 100 and 50 µm EASYstrainer cell strainers. To maximize protoplast recovery, 5–10 ml of chilled W5 solution [6,12] was used to rinse the filters. The filtrate was then centrifuged at 1,200*g* and 4 °C for 2 min. After discarding the supernatant, the pellet was resuspended in 5 ml of W5 solution by gently flicking the tube for several minutes. An additional 30 ml of W5 solution was added, and the suspension was centrifuged again at 1,200*g* and 4 °C for 2 min. The supernatant was discarded and the pellet was resuspended as before, and then gently pipetted up and down twice to ensure complete resuspension. The volume was then adjusted to 10 ml with W5 solution, and the protoplast suspension was kept on ice for up to 2 h before use.

To estimate the protoplast concentration, a 10 µl aliquot of the protoplast solution was transferred to a 2-Chip Disposable Haemocytometer (Bulldog Bio). The protoplasts were counted using an Olympus CKX53 microscope at 100× magnification with a Ph1 phase contrast ring (Olympus).

### PEG/CaCl_2_-mediated transformation

Transformations and mock transformations (see below) were conducted using the method described by Fang and Tyler [6] with minor modifications. For transformations, the protoplasts were centrifuged again at 1,200*g* and 4 °C for 2 min, and concentrations were adjusted to 2.5×10^7^ ml^−1^ in MMg solution [6,12]. For each transformation, we used 1 ml of protoplasts and 25 µg of plasmid DNA. PEG solution [6,12] was gradually added to the protoplasts in three successive 580 µl aliquots. The tube was gently rotated to evenly disperse the PEG solution, and between each aliquot, the tube was kept on ice for 2 min. After 20 min on ice, protoplasts were recovered in a total of 20 ml pea recovery medium through sequential additions of 2, 8 and 10 ml of recovery medium, incubating the protoplasts on ice for 2 min between each step. The protoplasts were then incubated in the dark at 22 °C for 16–18 h.

After recovery, germlings were pelleted at 2,000*g* for 5 min, and most of the liquid was gently discarded (leaving 200 µl–1 ml). For each transformation, 30 ml of pea-agarose recovery medium supplemented with antibiotics (50 µg ml^−1^ carbenicillin, 25–50 µg ml^−1^ G418, 50 µg ml^−1^ vancomycin) was added to the germlings. The tubes were inverted several times, and then the agarose was poured into 90 mm Petri dishes. The plates were incubated at 22 °C in the dark until transformants appeared.

After transformants appeared, a second round of selection was conducted, using one of two different approaches: (i) 6 mm agar plugs from the edge of colonies were transferred to pea broth supplemented with G418, or (ii) the transformation plate was overlaid with an additional 15 ml of molten recovery medium supplemented with G418. The concentration of G418 used in the secondary screen varied. Initially, 50 µg ml^−1^ was used; however, this was later reduced to 30 µg ml^−1^ G418 after observing some fluorescent transformants could not grow on media containing 50 µg ml^−1^ G418 media. The transformants were maintained on pea agar supplemented with 30 µg ml^−1^ G418.

### Mock transformations

A mock transformation procedure was used to optimize protoplast regeneration efficiency, where conditions were similar to the PEG/CaCl_2_-mediated transformation method described above but without the addition of DNA. For mock transformations, the regular procedure was also modified by pipetting protoplasts directly into different recovery media rather than adding recovery media to protoplasts sequentially. Four different recovery media and three different supplements were tested (i.e. clarified V8 broth, carrot broth, pea broth (all adapted from [27]) and a minimal media – each supplemented with either 25 µg ml^−1^ myo-inositol, 25 µg ml^−1^ lecithin, 25 µg ml^−1^ β-sitosterol or no added compound. The pH of the recovery media was adjusted using 1 M HCl or 5 M NaOH. Based on experimental results showing pH 5 as the optimal pH for recovery, all media in later experiments were standardized to this pH. All recovery media were stored at 4 °C until use. Aliquots of 5×10^5^ protoplasts were added to 2.5 ml of recovery medium, giving a final concentration of 2×10^5^ protoplasts per ml. Each tube was supplemented with antibiotics (50 µg ml^−1^ carbenicillin and 50 µg ml^−1^ vancomycin) and incubated horizontally at 22 °C in the dark for 16 h. Variable volumes of protoplasts were used; however, their concentration was standardized to 1×10^7^ protoplasts per ml and 1.74 ml of PEG 4,000 per 1 ml of protoplast solution.

After recovery, germlings were thoroughly resuspended by pipetting. When necessary, germlings were concentrated 10× before counting by pelleting at 2,000*g* for 5 min and discarding the supernatant until 10% of the original volume remained. The concentration of germlings was estimated using a haemocytometer. Images of each haemocytometer grid were taken at 40× magnification using a Ph1 phase contrast ring, and germlings were later counted manually. The recovery rate was estimated by dividing the observed germling concentration by the expected protoplast concentration and converting it to a percentage (i.e. multiplying by 100).

### PCR screening of transformants

pTdTomato transformants were verified by isolating their genomic DNA and subsequently amplifying the plasmid DNA by PCR. To isolate the genomic DNA, mycelial mats were grown in liquid potato dextrose broth for 5 days and then ground with liquid nitrogen into powder. This powder was then processed using the DNeasy Plant Mini Kit (Qiagen) [29]. Genomic DNA of both untransformed and transformed *P. agathidicida* were subsequently amplified with PCR primers targeting the *tdtomato* gene of the pTdTomato plasmid (Fw: 5′-ACA TCC CCG ATT ACA AGA AGC-3′; Rv: 5′-TTG TAG ATC AGC GTG CCG TC-3′).

### Production of oospores and zoospores from transformants

For oospore production, plugs of mycelia from cV8 plates were cultured at 22 °C in 15 ml liquid cV8 containing 10 µg ml^−1^ β-sitosterol in the dark for 120 h, which is similar to the method described by Lacey *et al.* [30], except for a reduced maturation time. Zoospores were produced using the method described by Lacey *et al.* [30] with minor modifications. Plugs of mycelia were cultured in liquid cV8 rather than carrot broth, and β-sitosterol concentration was decreased from 15 to 10 µg ml^−1^.

### Fluorescence microscopy

pTdTomatoN transformants were imaged directly in agar plates or Petri dishes of liquid media under varied conditions. To prepare pYF2-PsNLS-Cas9-GFP and pYF2-PsNLS-Cas12a-GFP transformants for imaging, hyphae were cultured on G418-supplemented media, washed in 25 ml sterile water and placed on glass microscope slides. Samples were then covered with 20 µl sterile water and a glass coverslip. Imaging was performed using an Olympus CKX53 microscope equipped with a U-RFL-T fluorescence unit. Green fluorescent protein (GFP) fluorescence was captured using a U-FBW mirror unit with excitation wavelengths of 460–495 nm, while TdTomato fluorescence was captured using a U-FGW mirror unit with excitation wavelengths of 530–550 nm. Exposure times varied, ranging from 400 to 800 ms for GFP-tagged proteins and 5–50 ms for TdTomato. Composite images were prepared in ImageJ 1.53 [31], using the *merge channels* function (under *Image>Colour*) to show brightfield images in greyscale and fluorescence images in red or green for TdTomato and GFP-tagged protein imaging, respectively.

### Data analysis

Analysis of protoplast data was carried out using R 4.2.2 [32]. For statistical analysis, model assumptions were assessed manually by inspecting residual-fitted value, normal quantile-quantile and residual-leverage plots. The statistical significance of the recovery results was assessed using an ANOVA. A multi-comparison post-test (Dunnett’s test) was used to compare individual treatments to the control.

## Results

### Protoplast isolation

**Fig. 1.**
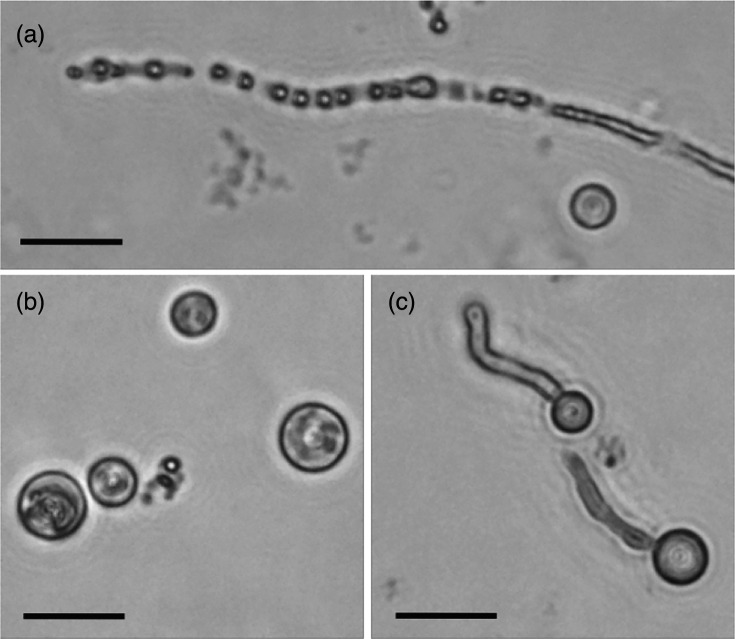
Formation and characteristics of *P. agathidicida* protoplasts. (**a**) Representative image showing the formation of protoplasts from a hypha via enzymatic digestion. Shown is an intact hypha (right side of the panel) as it is digested into protoplasts (left side of the panel). Protoplasts were digested using a mixture of 7.5 mg ml^−1^ (each) of cellulase from *Trichoderma sp*. and Lysing Enzymes from *T. harzianum*. (**b**) Representative image showing isolated protoplasts. (**c**) Representative image of protoplast germlings. The scale bars represent 20 µm.

Protoplast production from *P. agathidicida* was initially achieved using a mixture of cellulase from *Trichoderma sp*. and Lysing Enzymes from *T. harzianum* (each at 7.5 mg ml^−1^). This approach effectively digested *P. agathidicida* hyphae (Fig. 1a) within 45 min. Initially, small protoplasts (<10 µm) were observed, which subsequently swelled to 10–20 µm when resuspended in W5 solution (Fig. 1b). Some protoplasts were observed germinating after incubation in a recovery medium or osmotically stable buffer (e.g. MMg buffer, Fig. 1c). This germination indicated that a portion of the protoplasts remained viable and were suitable for transformation experiments.

**Fig. 2.**
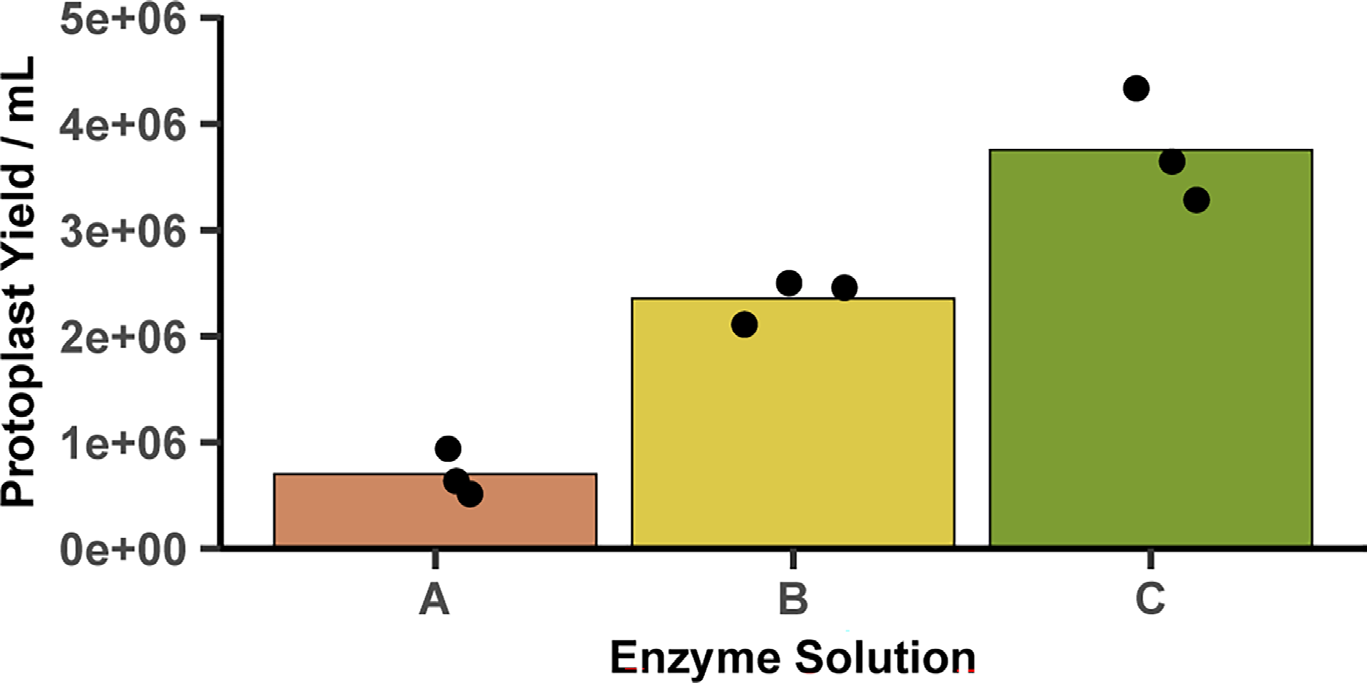
Effect of enzyme concentration on protoplast yields. Three enzyme solutions were tested. Solution A: 10 mg ml^−1^ cellulase *(Trichoderma sp*.) and 5 mg ml^−1^ Lysing Enzymes (*T. harzianum*). Solution B: 7.5 mg ml^−1^ cellulase *(Trichoderma sp*.) and 7.5 mg ml^−1^ Lysing Enzymes (*T. harzianum*). Solution C: 5 mg ml^−1^ cellulase *(Trichoderma sp*.) and 10 mg ml^−1^ Lysing Enzymes (*T. harzianum*). Bars represent the mean of independent biological triplicates (plotted as points; *n*=3).

Next, we aimed to maximize protoplast yield by varying the ratio of lysing enzymes and cellulase, keeping the total enzyme concentration constant. Three enzyme solutions were tested (Table 1). The protoplast yields obtained from the enzyme solution are shown in Fig. 2. The mean protoplast yield was highest in Solution C (3.8×10^6^ ml^−1^), containing a higher fraction of lysing enzyme than the other two solutions. Using Tukey’s test with a family-wise error rate of 0.05, there was strong evidence that the mean protoplast yield obtained with Solution C was greater than Solution B (2.4×10^6^ ml^−1^; *P=*0.0075) or Solution A (7.0×10^5^ ml^−1^; *P*<0.001).

### Effect of chitinase on protoplast yield

**Fig. 3.**
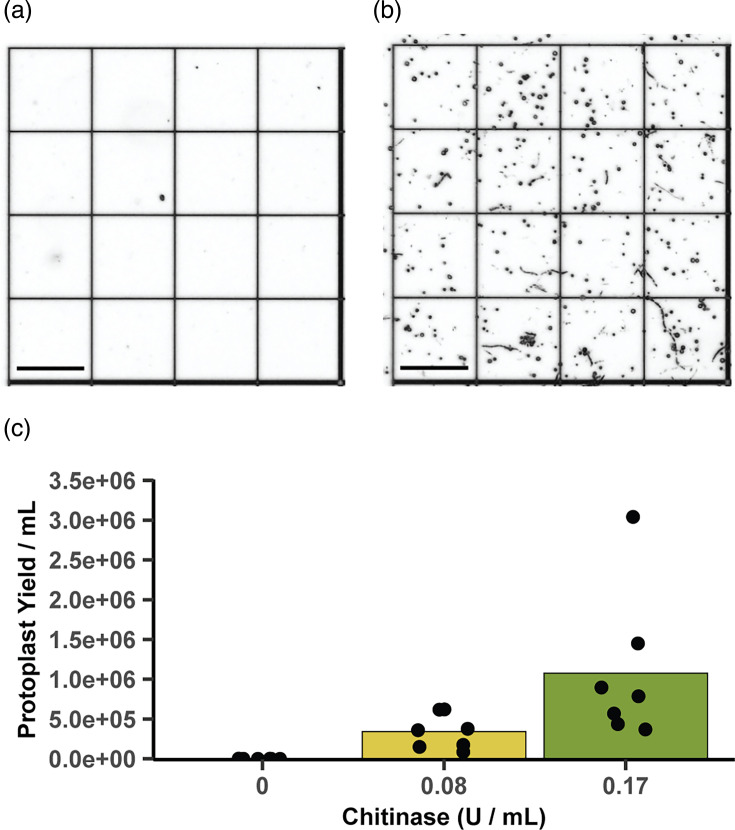
Effect of chitinase on protoplast yields. Panel (**a**) Representative image of protoplasts produced after 2 h of digestion with an enzyme cocktail of cellulase from *Trichoderma sp*. and β-glucanases from *Trichoderma longibrachiatum.* (**b**) Representative image of protoplasts produced after 2 h of digestion with cellulase from *Trichoderma sp*., β-glucanases from *T. longibrachiatum* and chitinase from *T. viride.* The scale bars in (a) and (b) represent 200 µm. (**c**) Comparison of protoplast yields obtained using an enzyme cocktail containing 5 mg ml^−1^ cellulase from *Trichoderma sp*., 10 mg ml^−1^ β-glucanases from *T. longibrachiatum* and either 0, 0.08 or 0.17 U ml^−1^ of chitinase from *T. viride.* Bars represent the mean of seven independent biological replicates (plotted as points; *n*=7).

Though the original enzyme cocktail used for protoplast formation (Table 1) was effective, one of the components – the Lysing Enzymes from *T. harzianum* – was subsequently discontinued in New Zealand. This led us to search for other enzymes that might be suitable substitutes. The original Lysing Enzymes mixture contained two main enzymes: β-glucanase and chitinase. For *Phytophthora* protoplast production, cellulases and β-glucanases are usually considered to be the only enzymes required for protoplast production [12,33]. Surprisingly, when we incubated *P. agathidicida* mycelia in a mixture of the same cellulase (5 mg ml^−1^) and β-glucanases from *T. longibrachiatum* (10 mg ml^−1^), almost no protoplasts were observed over a 3 h period (Fig. 3a), and even after 2 days, hyphae were partially intact (Fig. S1A).

Next, we supplemented the cellulase and β-glucanase mixture with chitinase from *T. viride* and observed that a large number of protoplasts were obtained within an hour (Figs 3b and S1B). To investigate this further, we tested chitinase-containing lysing enzyme mixtures with three different concentrations of chitinase (Table 2, Fig. 3c). Without chitinase in the lysing enzyme mixture, the yield of protoplasts was ≤1.1×10^3^ ml^−1^ (Fig. 3c). The yield increased to 3.4×10^5^ ml^−1^ when 0.08 U ml^−1^ chitinase was added and 1.0×10^6^ ml^−1^ with 0.17 U ml^−1^ of chitinase added (Fig. 3c). This increase in protoplast yield was statistically significant (0–0.17 U ml^−1^: t=−4.38, *P*<0.001; 0.08–0.17 U ml^−1^: t=−2.9, *P=*0.015).

### Effect of pH on protoplast recovery

**Fig. 4.**
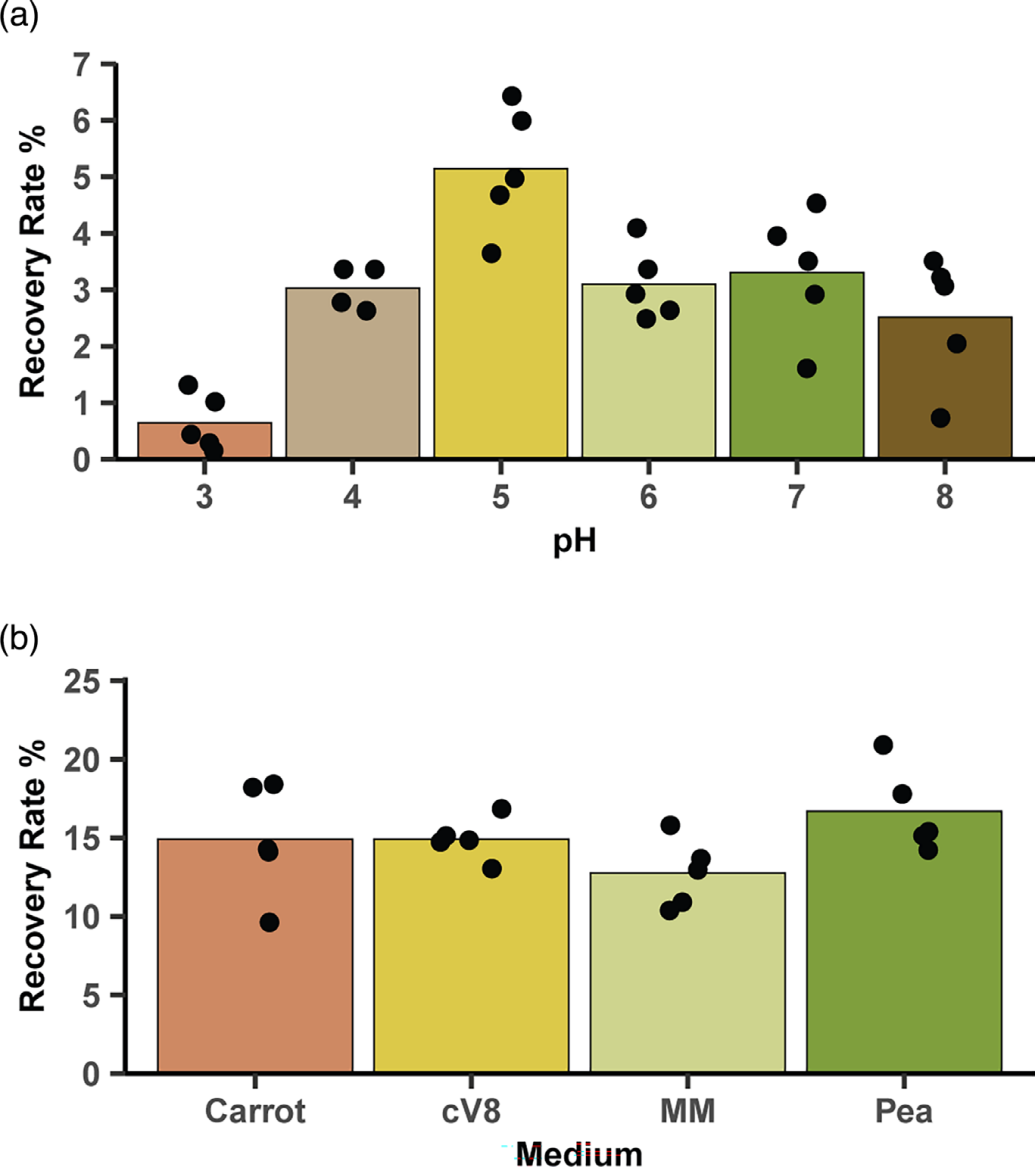
Effect of recovery media on protoplast regeneration. (**a**) The effect of pH on protoplast regeneration. Protoplasts were regenerated in pea recovery media at pH 3, 4, 5, 6, 7 or 8. (**b**) The effect of recovery medium composition on protoplast regeneration. Protoplasts were regenerated in carrot, clarified V8 (cV8), minimal medium (MM) or pea recovery media, all at pH 5. For both (**a**) and (**b**), bars represent the mean of five independent biological replicates (plotted as points; *n*=5).

Having developed a working protocol to produce protoplasts, we decided to optimize protoplast recovery. We first studied the effect of the recovery medium pH on *P. agathidicida* protoplast recovery rates; a set of six pea recovery media ranging from pH 3 to pH 8 were tested in mock transformations. Mean protoplast recovery at each pH was recorded (Fig. 4a). The maximum recovery was at pH 5, which significantly differed from pH 7 (t=3.3, *P*=0.014). We, therefore, used pH 5 for all our buffered solutions in subsequent transformations.

### Effect of media on protoplast recovery

We then decided to investigate the effect of different recovery media. We tested four recovery media: carrot, cV8, pea and a minimal recovery medium. Protoplasts were subjected to a ‘mock transformation’ procedure before aliquots were diluted into the various recovery media and allowed to recover. The percentage of protoplast recovery in each medium was calculated for three independent mock transformations (Fig. 4b).

Overall, the protoplast recovery rates were higher than in the pH experiment, probably due to improved handling of the protoplasts as we gained more experience. Using an ANOVA with a significance level of 0.05, no evidence of a difference in protoplast recovery rate was detected among the recovery media (F=1.9, *P*=0.17). We, therefore, selected the recovery medium with the highest mean recovery rate, which was the pea recovery medium (mean recovery=16.7 %). Additionally, we found that germlings generally grew longer in pea medium during a 16 h recovery period (Fig. S2).

We also investigated whether additional supplementary compounds could enhance protoplast recovery. We tested myo-inositol, l-α-phosphatidylcholine from soybean (lecithin) and β-sitosterol, all of which are known to support vigorous growth and/or sporulation in certain *Phytophthora* species [27]. We prepared 16 different recovery media by supplementing carrot, cV8, minimal and pea recovery media with either 25 µg ml^−1^ myo-inositol, 25 µg ml^−1^ lecithin, 25 µg ml^−1^ β-sitosterol or no supplement. Protoplast recovery rates were recorded in each medium for three independent mock transformations (Fig. S3). ANOVA was used to test for statistically significant differences in mean protoplast recovery rates within each set of four recovery media, with α=0.05. No differences in protoplast recovery rates were detected.

### Transformation of *P. agathidicida* protoplasts

After these optimization steps, we reattempted PEG/CaCl_2_-mediated protoplast transformations, scaling up the number of protoplasts to 2.5×10^7^ per transformation, with 25 µg of plasmid DNA. Here, we describe transformations using plasmids pTdTomatoN [28], pYF2-PsNLS-Cas9-GFP [6] and pYF2-PsNLS-Cas12a-GFP [5]. These plasmids express TdTomato fluorescent protein, *Streptococcus pyogenes* Cas9 nuclease (SpCas9) fused to the N-terminus of GFP and *Lachnospiraceae bacterium* Cas12a nuclease (LbCas12a) fused to the N-terminus of GFP, respectively. The Cas nuclease sequences both contain the PsNLS nuclear localization tag [6,34] at their N-terminus. The expression of these protein sequences is driven by the strong constitutive *ham34* promoter. All plasmids contain the *nptII* selectable marker gene, which encodes G418 (geneticin) resistance, but the promoter driving this gene differs: *nptII* is expressed from the *Hsp70* promoter in pTdTomatoN and from the *rpl41* promoter in the other plasmids.

**Fig. 5.**
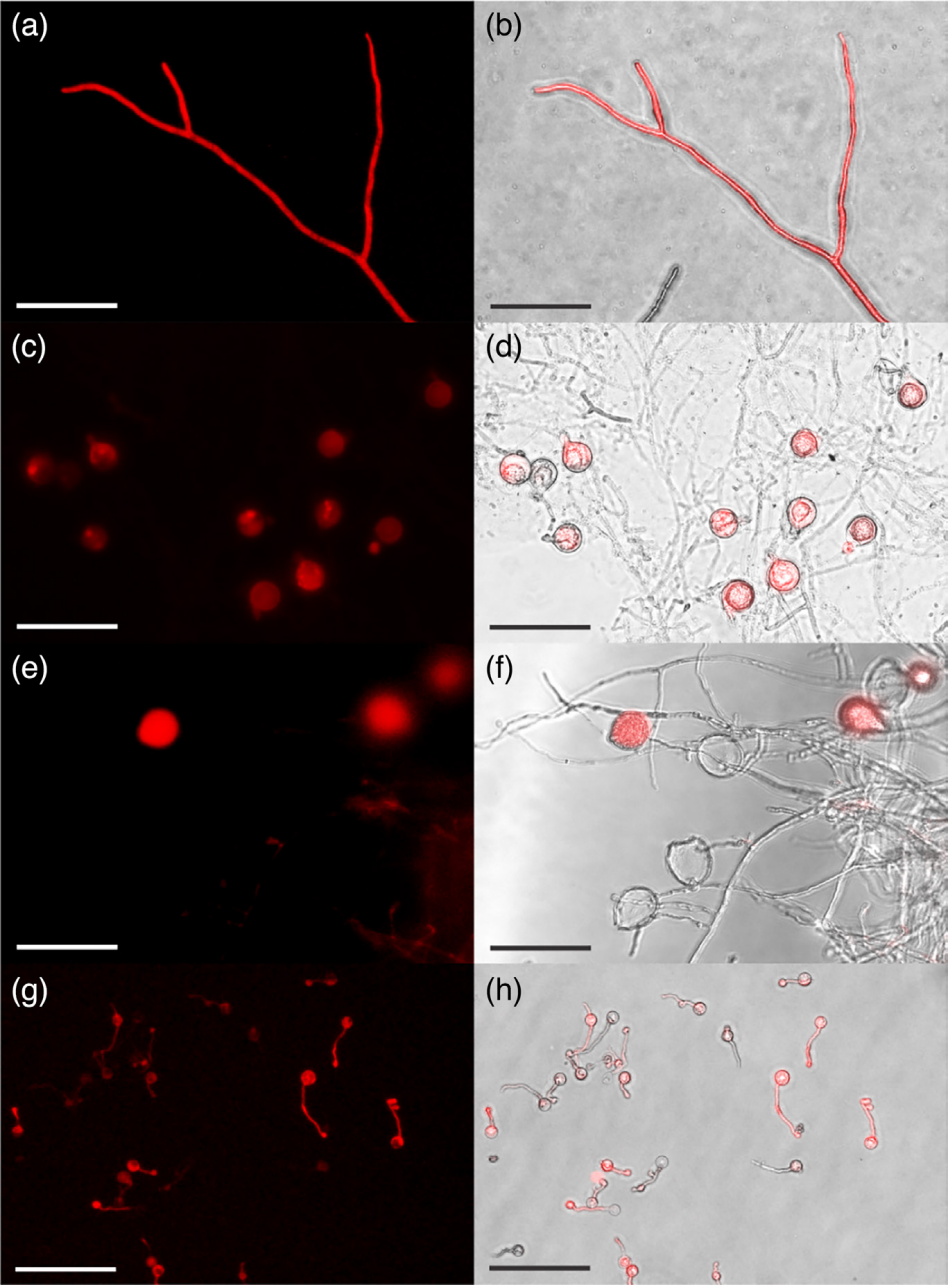
Key lifecycle stages (i.e*.* hyphae, oospores, sporangia and cysts) of pTdTomatoN transformants exhibiting fluorescence. Panels (**a**) and (**b**) show growing hyphae in agar, where (**a**) is a representative fluorescence image and (**b**) is the merged image of brightfield and fluorescence. Panels (**c**) and (**d**) show oospores and old hyphae, where (**c**) is a representative fluorescence image and (**d**) is the merged brightfield and fluorescence. Panels (**e**) brightfield and (**f**) fluorescence show sporangia and hyphae. Panels (**g**) brightfield and (**h**) show germinating cysts. The scale bars represent 50 µm.

Transformation with pTdTomatoN was performed in two independent experiments. Transformants that exhibited growth on selective media following the second round of G418 selection were classified as transformants. Based on this criterion, two transformants were obtained in the first round and three in the second. We observed that the fluorescence of TdTomato varied among the transformants but remained stable when they were grown on media containing G418 for the duration of the study, which was at least 8 weeks. Fluorescence was observed in all life cycle stages of the transformants: hyphae (Fig. 5a, b), oospores (Fig. 5c, d), sporangia (Fig. 5e, f) and germinating cysts (Fig. 5g, h). The fluorescent signal was strong enough to be seen directly in agar plates. In contrast, no substantial fluorescence was observed in wild-type *P. agathidicida* cultures, even when the exposure was increased (Figs S4, S5 and S6). Fluorescence was brightest in actively growing hyphal tips, sporangia and oospores. Old hyphal growth (Fig. 5c, d) and empty sporangia (Fig. 5e, f) did not show a fluorescent signal. A PCR reaction using genomic DNA extracted from a pTdTomatoN transformant with *tdtomato*-specific primers yielded a product of the correct size (Fig. S7), confirming the transformant harboured the *tdtomato* gene. When we attempted to culture transformants in media without G418, the fluorescence was lost within approximately one week, though it could be recovered by reintroducing the transformant to G418-selective media.

**Fig. 6.**
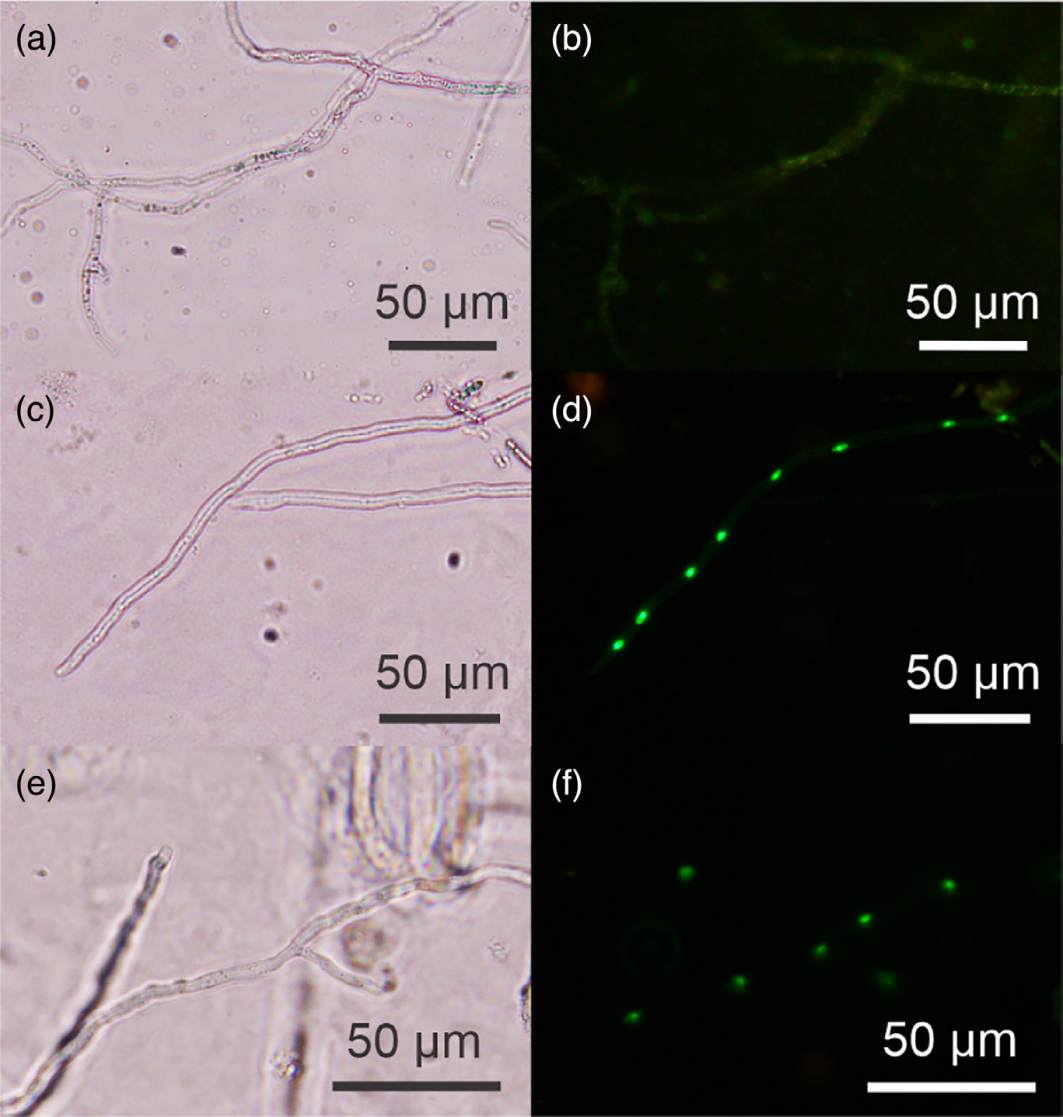
Comparison of wild-type *P. agathidicida* and the pYF2-PsNLS-dCas9-GFP and pYF2-PsNLS-Cas12a-GFP transformants. Representative wild-type *P. agathidicida* hyphae (**a**) brightfield and (**b**) fluorescence images. PsNLS-pCas9-GFP transformant hyphae (**c**) brightfield and (**d**) fluorescence image showing localization of the protein to nuclei within a hypha. PsNLS-Cas12a-GFP (**e**) brightfield and (**f**) fluorescence image showing localization of the protein to nuclei within a hypha. The scale bars represent 50 µm.

In addition to cytoplasmic labelling, we wanted to visualize the expression of Cas nucleases in *P. agathidicida* to verify expression and nuclear localization, which are key to successful genome editing experiments [5,7]. We attempted transformation three times using pYF2-PsNLS-Cas9-GFP (yielding five, five and three transformants) and once with pYF2-PsNLS-Cas12a-GFP (this yielded six transformants). To assess Cas nuclease-GFP expression, we compared fluorescence in wild-type *P. agathidicida* hyphae to putative pYF2-PsNLS-Cas9-GFP and pYF2-PsNLS-Cas12a-GFP transformants (Fig. 6). In wild-type *P. agathidicida*, faint green autofluorescence was seen throughout most hyphae (Fig. 6a, b). We observed markedly brighter fluorescence localized in points showing localization of the protein to nuclei within the hyphae (generally at hyphal tips) in the pYF2-PsNLS-Cas9-GFP transformants (Fig. 6c, d) and pYF2-PsNLS-Cas12a-GFP transformants (Fig. 6e, f) but not untransformed hyphae (Fig. 6a, b). Visible fluorescence from GFP-tagged Cas nucleases was lost within a week after the second round of selection, so we could not detect GFP fluorescence in any other life cycle stage.

## Discussion

### The importance of chitinase for *P. agathidicida* protoplast formation

In this study, we have demonstrated for the first time that *P. agathidicida* is amenable to protoplasting and PEG/CaCl_2_-mediated protoplast transformation. We found that an enzyme mixture consisting of 10 mg ml^−1^ Lysing Enzymes from *T. harzianum* and 5 mg ml^−1^ cellulase from *Trichoderma sp*. was effective for protoplast production. We also investigated β-glucanase from *T. longibrachiatum* and chitinase from *T. viride* as alternatives to the Lysing Enzymes product. Interestingly, chitinase activity appears to be a crucial ingredient for the production of *P. agathidicida* protoplasts. Although oomycetes are known to possess chitin synthases [35,37], chitin is not thought to be abundant in the hyphae of *Phytophthora infestans* and *Phytophthora parasitica* [36,38]. Whether *P. agathidicida* has a higher chitin content than these previously studied *Phytophthora* species remains to be determined. To our knowledge, there are no previously published protocols that specifically use chitinases to generate *Phytophthora* protoplasts. However, chitinase is a component of the commercial Lysing Enzymes cocktail from * T. harzianum*. Nevertheless, we observed a major reduction in protoplast yield (from 10^6^ ml^−1^ to ~10^3^ ml^−1^ when chitinase was omitted, suggesting that chitin linkages (specifically, *N*-acetyl-β-d-glucosamine (1→4)-β-linkages) are present in the cell walls of *P. agathidicida* hyphae. It would be valuable to explore whether chitinase activity also aids protoplast formation in other oomycetes, though we cannot rule out the possibility that the commercially available chitinase from *T. viride* exhibits other enzymatic activities targeting carbohydrate linkages in *Phytophthora* species, such as glucuronic acid [38]. Regardless, variations of this enzyme mixture might prove useful to the wider oomycete community, especially those in countries where Lysing Enzymes from *T. harzianum* or other formulations such as Extralyse are unavailable.

### Factors affecting protoplast recovery

After initially struggling to obtain *P. agathidicida* transformants, we set out to optimize the recovery medium and identify variables that might influence success. Our results showed that the pH of the recovery medium has a statistically significant impact on recovery, with an optimum pH of approximately five for *P. agathidicida* protoplasts; this is similar to the pH of kauri forest soil [39]. Surprisingly, the recovery media base (e.g. carrot and pea) was not a statistically significant predictor of protoplast recovery. Similarly, when we supplemented the recovery media with compounds known to aid growth and/or sporulation in other *Phytophthora* species [27], we did not observe any significant changes in protoplast recovery. However, it remains possible that different concentrations or additional compounds could impact protoplast recovery. We continued to use the pea medium because it gave the highest mean recovery rate, and germlings grew longer in the pea medium than the others over 16 h; however, we did not measure this directly.

Overall, aside from pH, the composition of the recovery medium does not appear to be a significant variable for *P. agathidicida* protoplast regeneration.

### Transformation of *P. agathidicida*

We expressed three fluorescent proteins in *P. agathidicida*: TdTomato [28] in the cytoplasm, SpCas9-GFP [6] in nuclei and LbCas12a-GFP [5] in nuclei. Collectively, these results showed that three common oomycete promoter sequences (*ham34*, *rpl41* and *hsp70*) are all functional in *P. agathidicida*. Similarly, the PsNLS sequence used to localize Cas nucleases to nuclei in other *Phytophthora* species [5,6] was also sufficient for nuclear localization in *P. agathidicida*.

Maintaining expression of the fluorescent marker in pTdTomatoN transformants required the continuous presence of G418; transgene expression was lost in the absence of selective pressure. Similarly, fluorescence of GFP-tagged Cas nucleases was not observed beyond 1 week after the second round of G418 selection, though the transformants retained G418 resistance. This instability without selective pressure aligns with previous observations in other *Phytophthora* species [40,44]. Despite the need for continuous antibiotic selection to maintain transgene expression, these transformants remain valuable for various applications. Importantly, transient expression systems are sufficient for many cell-tracking and chemotaxis studies, as well as CRISPR-Cas genome editing approaches. The observed fluorescence of SpCas9-GFP and LbCas12a-GFP fusion proteins indicated relatively high expression levels and successful nuclear localization. These findings are particularly promising for future CRISPR-Cas genome editing efforts in *P. agathidicida*, demonstrating that even transient expression can potentially provide adequate Cas nuclease activity for targeted genetic modifications. Furthermore, the ability to track cells using fluorescent markers like TdTomato will enhance our understanding of *P. agathidicida*’s lifecycle and behaviour.

We also noted that transformation with the pCas9-GFP and pCas12a-GFP plasmids generally yielded more transformants than the pTdTomatoN plasmid, and recovery of pTdTomatoN transformants was generally slower. One possible explanation is that the *rpl41* promoter that drives *nptII* expression in the Cas nuclease-GFP plasmids is more active in *P. agathidicida* than the *Hsp70* promoter in the pTdTomatoN plasmid. Thus, it might be worthwhile to test the activity of different promoter sequences in *P. agathidicida*. This could be done using a β-glucuronidase reporter assay, which has been used to assess different promoters in *P. infestans* [45]. Another abnormality we noted in transformants was that sporulation was generally lower on G418-supplemented media. Sporulation appeared normal while on non-selective media but could not be restored when returned to selective media – a phenomenon also reported in *P. parasitica* [41]. As such, it would be useful to explore whether other selection markers are better suited to *P. agathidicida*, for example, hygromycin [8], oxathiapiprolin [44] or gentamicin [46].

Additionally, there are several avenues for optimizing transformation that we did not explore. The effect of lipofectin on transformation efficiency in *P. agathidicida* could be tested. Lipofectin increases transformation efficiency in *P. infestans* but is also costly and subject to significant batch-to-batch variation [33]. Another option is to test whether using cultures containing a higher percentage of young growth increases the recovery rate and/or transformation frequency in the resulting protoplasts. In some oomycetes, such as *P. infestans*, the sporangia are detachable (caducous) and are the preferred inoculum for transformation [8]. This is not feasible for *P. agathidicida*, which has non-caducous sporangia [16]. However, *P. agathidicida* can produce substantial numbers of zoospores, which could be germinated and used to produce protoplasts [22,25]. Finally, it would be worthwhile to test whether different isolates of *P. agathidicida* are more amenable to protoplast transformation. Strain variability has been noted for several transformation methods in oomycetes [47,48] and may provide a simple route to increased transformation frequency. However, at present, only isolate NZFS 3770 has a chromosome-level genome sequence available [20], so it remains the best candidate for functional genomic experiments such as gene silencing or knockouts.

This research has demonstrated that *P. agathidicida* is amenable to PEG/CaCl_2_-mediated protoplast transformation, enabling the transient expression of fluorescent proteins and Cas nucleases. Although the transformants require antibiotic selection for stability, this method provides a valuable tool for studying this plant pathogen. However, there remains scope for further refinement, particularly in achieving stable transformation. Future research should focus on investigating mechanisms affecting transgene stability, including heterokaryosis, chromosomal integration and gene silencing. For example, stability may be enhanced by creating mononucleate lines from the obtained transformants through single zoospore passaging, which can overcome the issue of heterokaryon transformations [41,43, 49, 50]. Other factors to consider include gene silencing [42,51] and potential episomal replication of transgenes [11,52, 53]. Further study is needed to investigate the conditions under which these events occur and design strategies to minimize them.

Overall, this study lays the groundwork for optimizing transformation techniques in *P. agathidicida* and potentially other Clade 5 *Phytophthora* species. By establishing molecular biology tools for *P. agathidicida*, this work opens up several avenues for further research exploring the biology of this important plant pathogen.

## supplementary material

10.1099/mic.0.001547 Uncited Supplementary Material 1.

## Abbreviations

CaCl_2_: calcium chloride
CRISPR: clustered regularly interspaced short palindromic repeats
gDNA: genomic DNA
GFP: green fluorescent protein
SpCas9: Streptococcus pyogenes Cas9 nuclease
