# Supplementary material for: Transient expression of fluorescent proteins and Cas nucleases in Phytophthora agathidicida via PEG-mediated protoplast transformation
Source: Microbiology (Reading). 2025 Mar 28;171(3):001547. doi: 10.1099/mic.0.001547 (PMC11952662; doi:10.1099/mic.0.001547)
Supplement: Uncited Supplementary Material 1. [file mic-171-01547-s001.pdf]

Supplementary Material for: Transient expression of fluorescent proteins and Cas nucleases in *Phytophthora agathidicida* via PEG-mediated protoplast transformation

Max Hayhurst[1,#], Jochem NA Vink[1#], Maxence Remerand[1], and Monica L Gerth[1*]

[1]School of Biological Sciences, Victoria University of Wellington, New Zealand.

*Corresponding author: monica.gerth@vuw.ac.nz

Supplementary Materials and Methods

Supplemental stock solutions: solutions of the supplemental compounds were prepared at 100× concentration in either water (for myo-inositol, lecithin) or absolute ethanol (for β-sitosterol).

The following recipes were adapted from (Mcleod et al., 2008) and (Fang et al., 2017; Fang & Tyler, 2016):

W5 solution consisted of 5 mM KCl, 125 mM $CaCl_2$, 154 mM NaCl, and 173 mM D-glucose.

Minimal recovery medium was made from yeast nitrogen base without amino acids (Sigma-Aldrich) at 1× concentration and supplemented with 2% (w/v) D-glucose.

20% (w/v) pea medium (agar or broth) was prepared by blending frozen peas (20% w/v) in ultrapure water (filled to 80% of the final desired volume). Solid debris was pelleted by centrifugation at 7,000*g* for 20 min, and the supernatant was filtered through Whatman® grade 1 filter paper. Ultrapure water was added to 1 L along with bacteriological grade agar before sterilising by autoclaving. For liquid medium, bacteriological agar was excluded. All growth media were stored at 4 °C.

Pea recovery medium was prepared by supplementing 20% (w/v) pea broth (before autoclaving) with 0.5 M D-mannitol, 10 mM $CaCl_2$ and 2 g/L $CaCO_3$. The pH was adjusted using 1 M HCl (for the final optimised protocol, this was pH 5). Solids were pelleted by centrifugation at 7,000*g* for 10 min. The liquid was decanted into a bottle and sterilised by autoclaving. Precipitate may form during autoclaving, but a clear recovery medium can be obtained by decanting into a fresh bottle or tube.

Pea-agarose recovery medium was prepared by adding low melting point agarose to a final concentration of 2% (w/v) to pea recovery medium. After autoclaving, it was cooled to ~30-35 °C before use.

The following recipes were adapted from (Erwin & Ribeiro, 1996):

20% (w/v) carrot broth comprised 20% (w/v) frozen baby carrots. The carrots were blended with water, then the solids were pelleted by centrifugation at 7,000*g* for 10 min. The liquid was decanted into a bottle and sterilised by autoclaving. Adapted from

20% (v/v) cV8 broth was composed of 20% V8 juice. $CaCO_3$ (10 g/L) was added to undiluted V8 juice, and the solution was stirred for 15 min before centrifuging at 7,000g for 10 min. The supernatant (clarified broth) was decanted and diluted to 20% (v/v) with water, and the clarified broth was then sterilised by autoclaving.

2% (w/v) soil solution was made from autoclaved, dried soil collected at Kelburn Park (Wellington, New Zealand). The solution was prepared by adding 20 g soil to 1 L ultrapure water, mixing thoroughly for 2 h, pelleting solid material by centrifugation (7,000*g* for 30 min), and passing the supernatant through Whatman® grade 1 filter paper. The solution was autoclaved and stored at room temperature until use.

References

Erwin, D. C., & Ribeiro, O. K. (1996). *Phytophthora diseases worldwide*. The American Phytopathological Society (APS Press).

Fang, Y., Cui, L., Gu, B., Arredondo, F., & Tyler, B. M. (2017). Efficient genome editing in the oomycete *Phytophthora sojae* using CRISPR/Cas9. *Current Protocols in Microbiology*. https://doi.org/DOI: 10.1002/cpmc.25

Fang, Y., & Tyler, B. M. (2016). Efficient disruption and replacement of an effector gene in the oomycete *Phytophthora sojae* using CRISPR/Cas9. *Molecular Plant Pathology*, *17*(1), 127-139.

Mcleod, A., Fry, B. A., Zuluaga, A. P., Myers, K. L., & Fry, W. E. (2008). Toward improvements of Oomycete transformation protocols. *Journal of Eukaryotic Microbiology*, *55*(2), 103-109. https://doi.org/https://doi.org/10.1111/j.1550-7408.2008.00304.x

Supplementary Figures

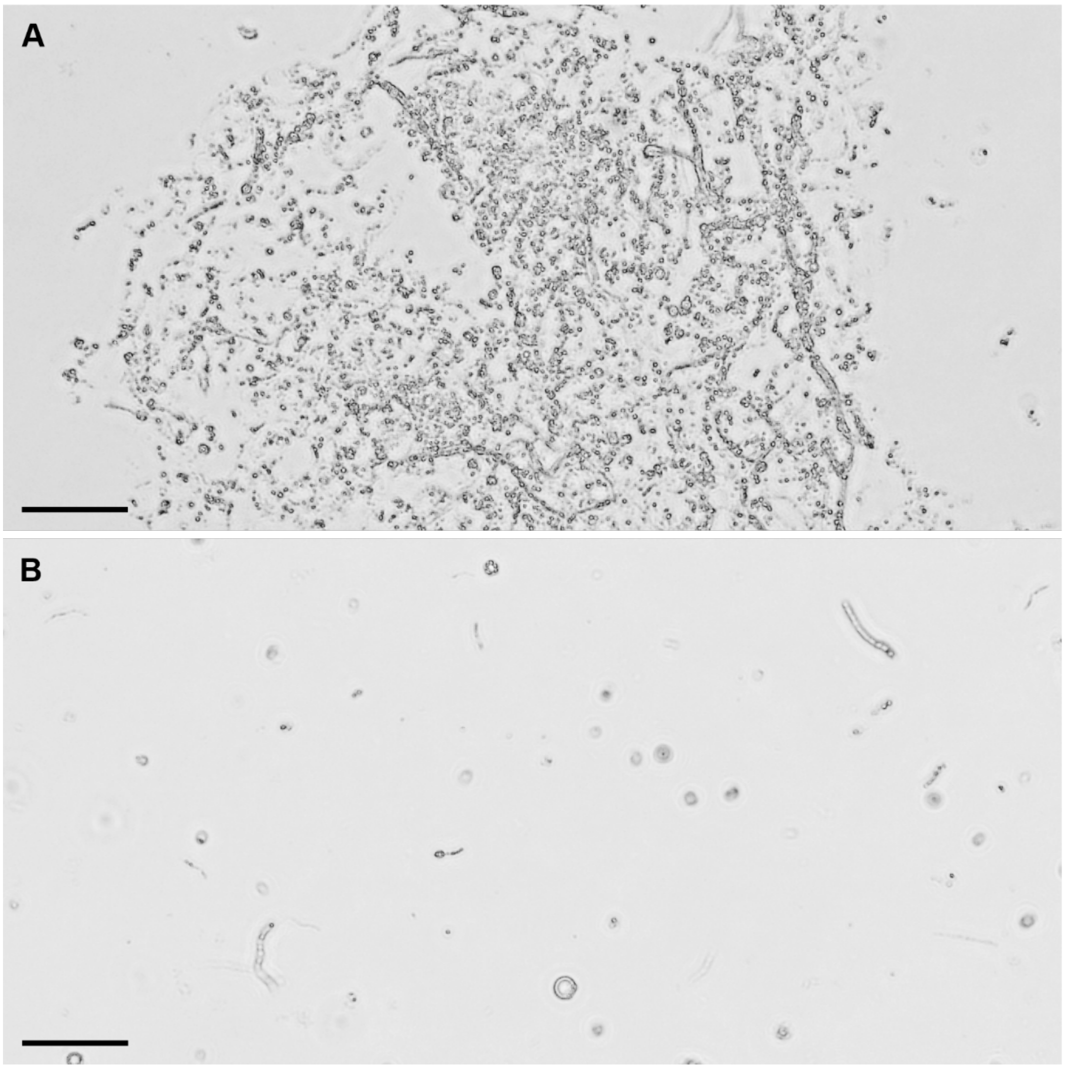

Figure S1. Representative images of protoplast formation using enzyme solutions with or without chitinase. (A): Hyphal debris after 44 h digestion in the β-glucanase and cellulase digesting enzyme solution. (B): 1 h digestion in the β-glucanase, cellulase, and chitinase digesting enzyme solution. The scale bars represent 20 μm.

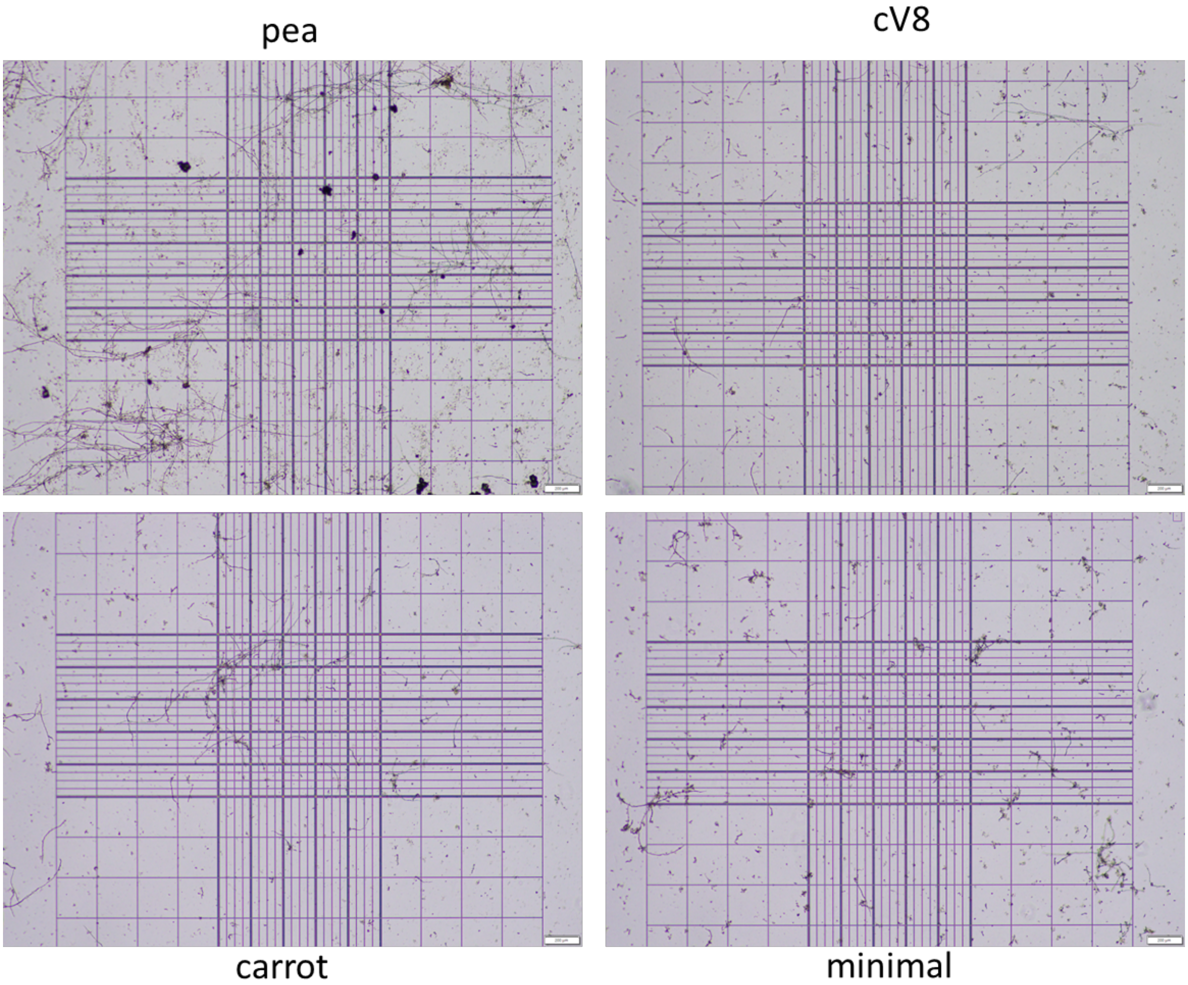

Figure S2. Representative images of germinated protoplasts after 16 h recovery.
(A): Germlings incubated in pea recovery medium; (B): germlings incubated in cV8 recovery medium; (C): germlings incubated in carrot recovery medium; (D): germlings incubated in minimal recovery medium.

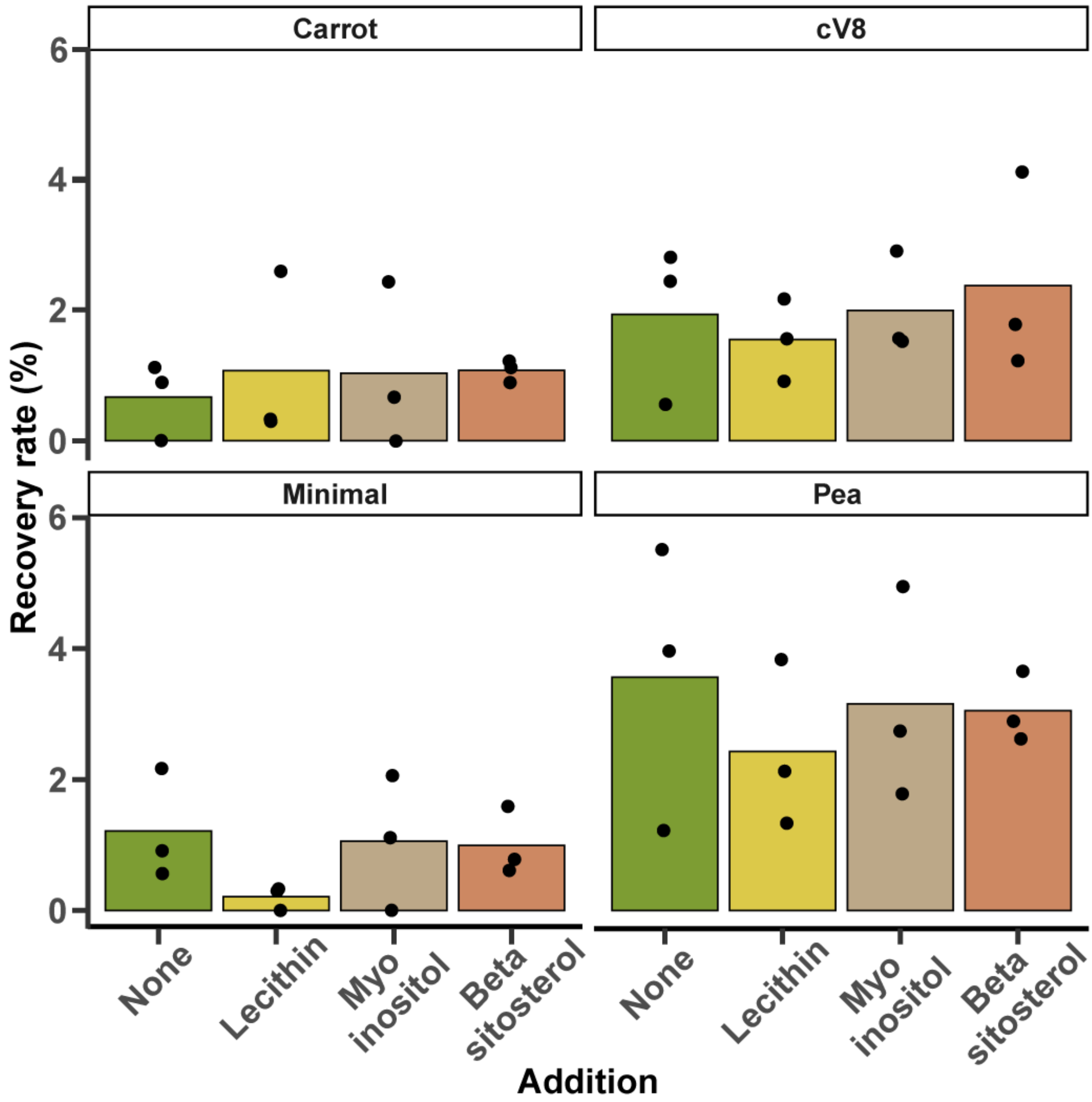

Figure S3: Protoplast recovery rates in recovery media supplemented with selected compounds. (A) Recovery rates in carrot recovery medium. (B) Recovery rates in cV8 recovery medium. (C) Recovery rates in minimal recovery medium. (D) Recovery rates in pea recovery medium. Protoplast recovery rates are plotted as a percentage (Y axes) and supplemental compounds are shown on the X axes. Bars represent the mean recovery rate from three independent biological replicates, which are plotted as individual datapoints.

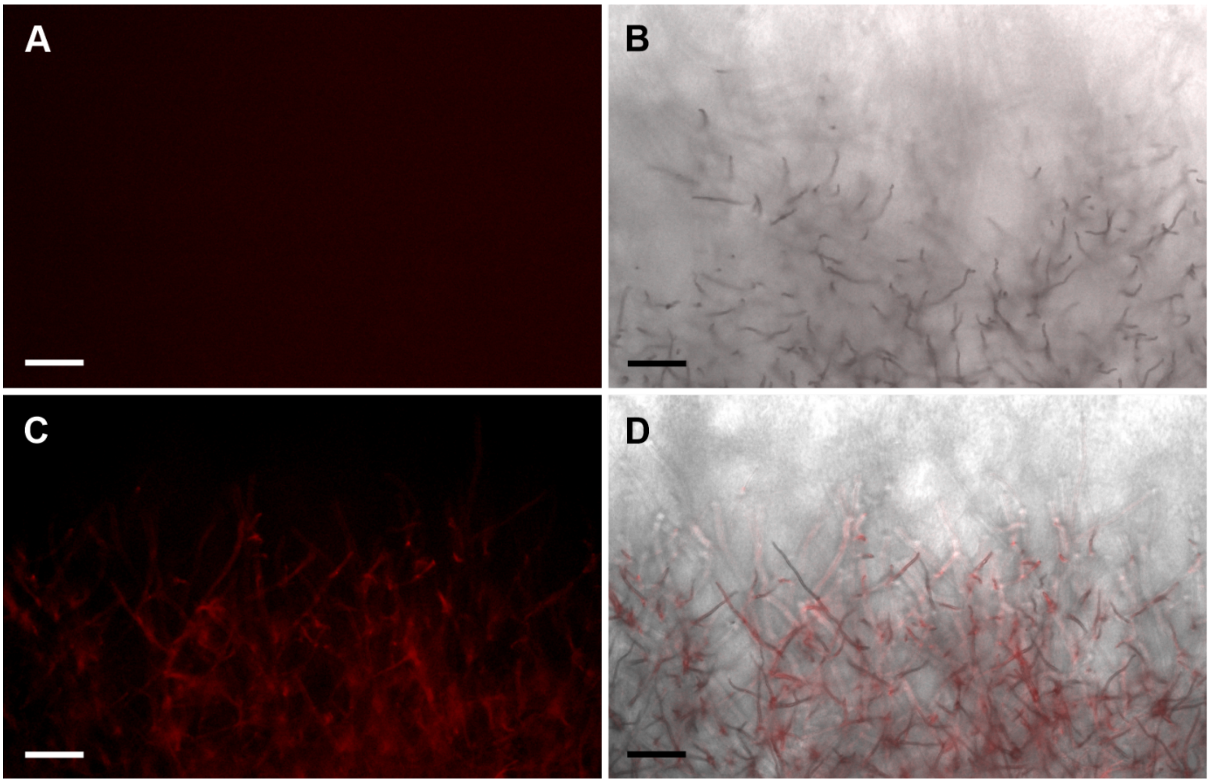

Figure S4. Fluorescence in wild type and pTdTomatoN transformant hyphae. Images were either captured using a red filter (A, C) or are composites of the red filter and brightfield view (B, D). From top to bottom, the images show wild-type *P. agathidicida* hyphae in agar (A, B) and pTdTomatoN transformant hyphae in agar (C, D). All scale bars (black or white lines, bottom left corner of each image) represent 50 μm.

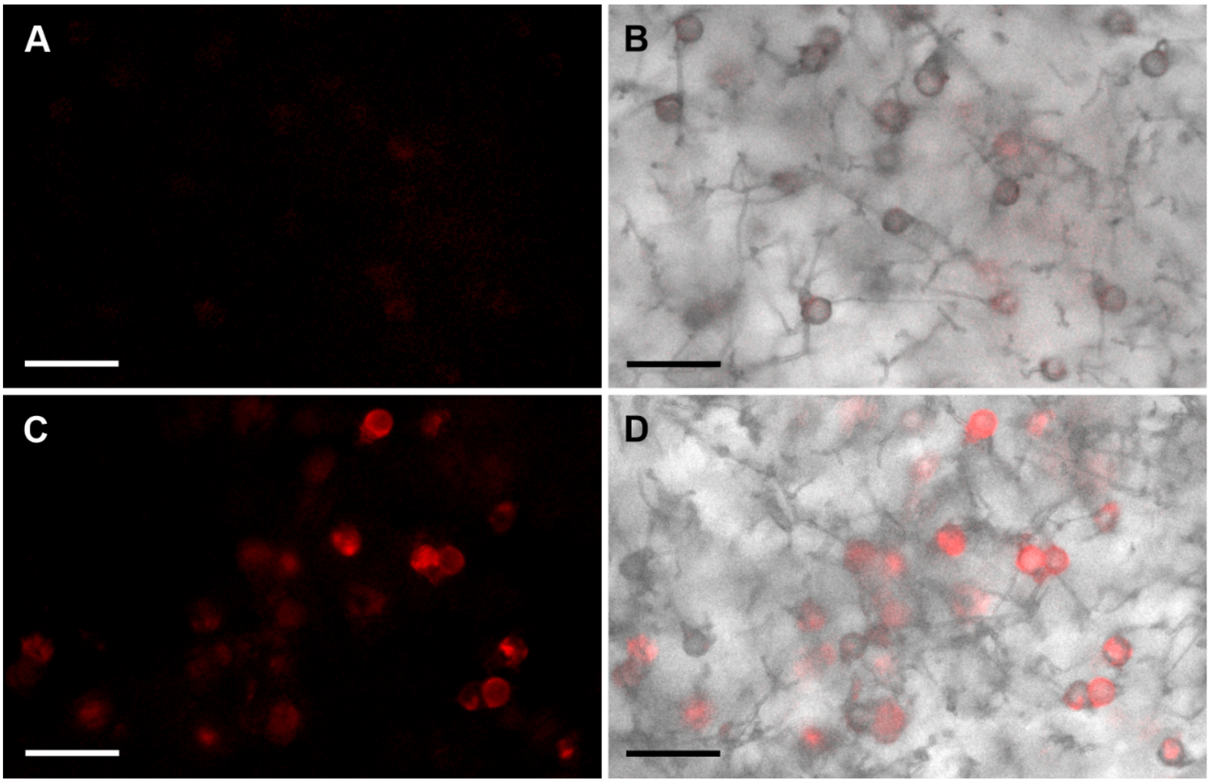

Figure S5. Fluorescence in wild type and pTdTomatoN transformant oospores. Images were either captured using a red filter (A, C) or are composites of the red filter and brightfield view (B, D). From top to bottom, the images show: wildtype *P. agathidicida* oospores and old-growth hyphae in agar (A, B); pTdTomatoN transformant oospores and old-growth hyphae in agar (C, D). All scale bars (black or white lines, bottom left corner of each image) represent 100 µm.

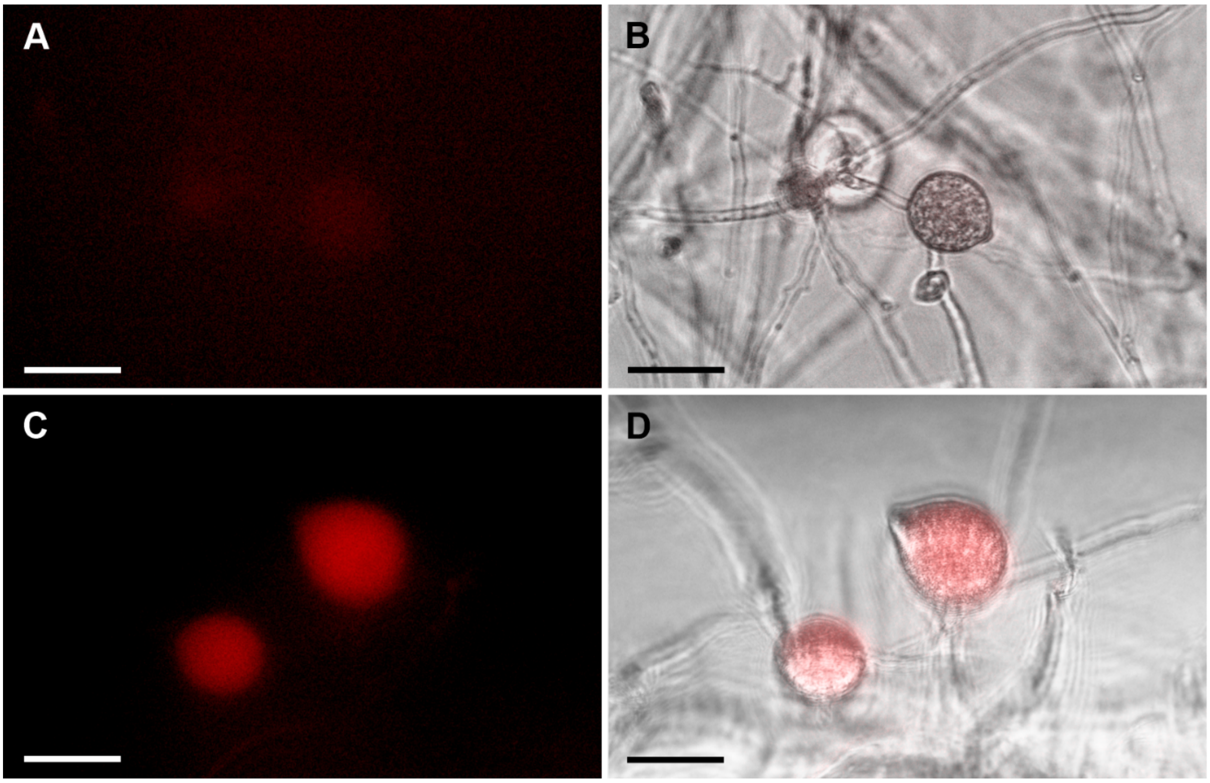

Figure S6. Fluorescence in wild type and pTdTomatoN transformant sporangia. Images were either captured using a red filter (A, C) or are composites of the red filter and brightfield view (B, D). From top to bottom, the images show: wild type *P. agathidicida* sporangia and hyphae in water (A, B); pTdTomatoN transformant sporangia and hyphae in water (C, D). All scale bars (black or white lines, bottom left corner of each image) represent 40 µm.

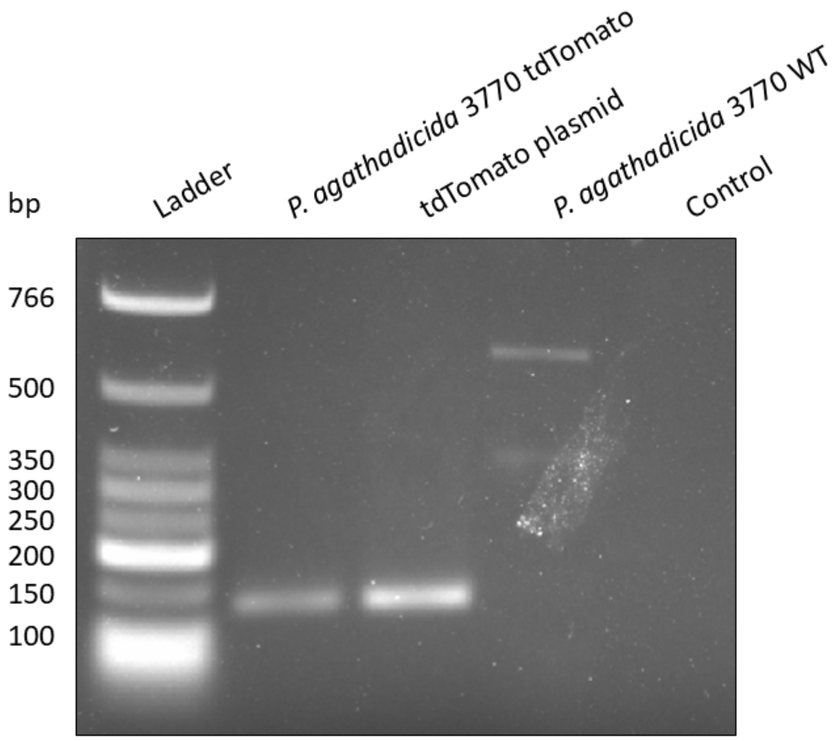

Figure S7. PCR screen for *tdtomato* gene in transformant. The agarose gel lanes contain DNA from PCR reactions using primers targeting the *tdtomato* gene. Templates for the PCRs were gDNA from a pTdTomatoN transformant (2nd lane), purified pTdTomatoN plasmid (3rd lane), gDNA form *P. agathidicida* wild type (4th lane) and a negative control (no DNA; 5th lane). The ladder (1st lane) is a Low Molecular Weight DNA Ladder (New England Biolabs).
